# Assessment of the potential risk of oteseconazole and two other tetrazole antifungals to inhibit adrenal steroidogenesis and peripheral metabolism of corticosteroids

**DOI:** 10.3389/fphar.2024.1394846

**Published:** 2024-08-08

**Authors:** Marie-Christin Jäger, Víctor González-Ruiz, Friedrich L. Joos, Denise V. Winter, Julien Boccard, Thorsten Degenhardt, Steve Brand, Serge Rudaz, George R. Thompson, Alex Odermatt

**Affiliations:** ^1^ Swiss Centre for Applied Human Toxicology (SCAHT), University of Basel, Basel, Switzerland; ^2^ Division of Molecular and Systems Toxicology, Department of Pharmaceutical Sciences, University of Basel, Basel, Switzerland; ^3^ Institute of Pharmaceutical Sciences of Western Switzerland, University of Geneva, Geneva, Switzerland; ^4^ Mycovia Pharmaceuticals Inc., Imperial Business Park, Durham, NC, United States; ^5^ Department of Internal Medicine, Division of Infectious Diseases, University of California–Davis Health, Sacramento, CA, United States

**Keywords:** azole antifungal, steroidogenesis, adverse drug reaction, cytochrome P450, H295R, steroid profile, enzyme, inhibition

## Abstract

The triazole antifungals posaconazole and itraconazole can cause pseudohyperaldosteronism with hypertension and hypokalemia, edema, and gynecomastia by inhibiting steroid synthesis and metabolism. Mechanisms underlying pseudohyperaldosteronism include inhibition of adrenal 11β-hydroxylase cytochrome-P450 (CYP) 11B1 and 17α-hydroxylase (CYP17A1) as well as peripherally expressed 11β-hydroxysteroid dehydrogenase type 2 (11β-HSD2). To enhance specificity for fungal CYP51, tetrazoles have been developed. This study employed H295R adrenocortical cells and enzyme activity assays to assess the potential risk of oteseconazole and two other tetrazoles, VT-1598 and quilseconazole, to inhibit adrenal steroidogenesis or 11β-HSD2. Steroidomic footprint analyses of H295R cell supernatants using untargeted liquid-chromatography-high-resolution mass-spectrometry (LC-HRMS) indicated overall patterns common to oteseconazole, quilseconazole and itraconazole, as well as similarities between VT-1598 and isavuconazole. Additionally, more specific features of the steroid signatures were observed. Targeted quantification of nine adrenal steroids in supernatants from treated H295R cells revealed an overall inhibition of adrenal steroidogenesis by the three tetrazoles, itraconazole and isavuconazole, providing an explanation for their similar steroidomic pattern. Applying recombinant enzymes indicated that this effect is not due to direct inhibition of steroidogenic enzymes because no or only weak inhibition could be observed. Moreover, oteseconazole and the two other tetrazoles did not inhibit 11β-HSD2, suggesting that they do not pose a risk of pseudohyperaldosteronism. Furthermore, oteseconazole did not alter steroid concentrations in a recent clinical study. Nevertheless, follow-up studies should assess the mechanism underlying the observed overall steroidogenesis inhibition by tetrazoles, itraconazole and isavuconazole, and whether concentrations achievable in a subgroup of susceptible patients might cause adrenal insufficiency and hyperplasia.

## 1 Introduction

**FIGURE 1:**
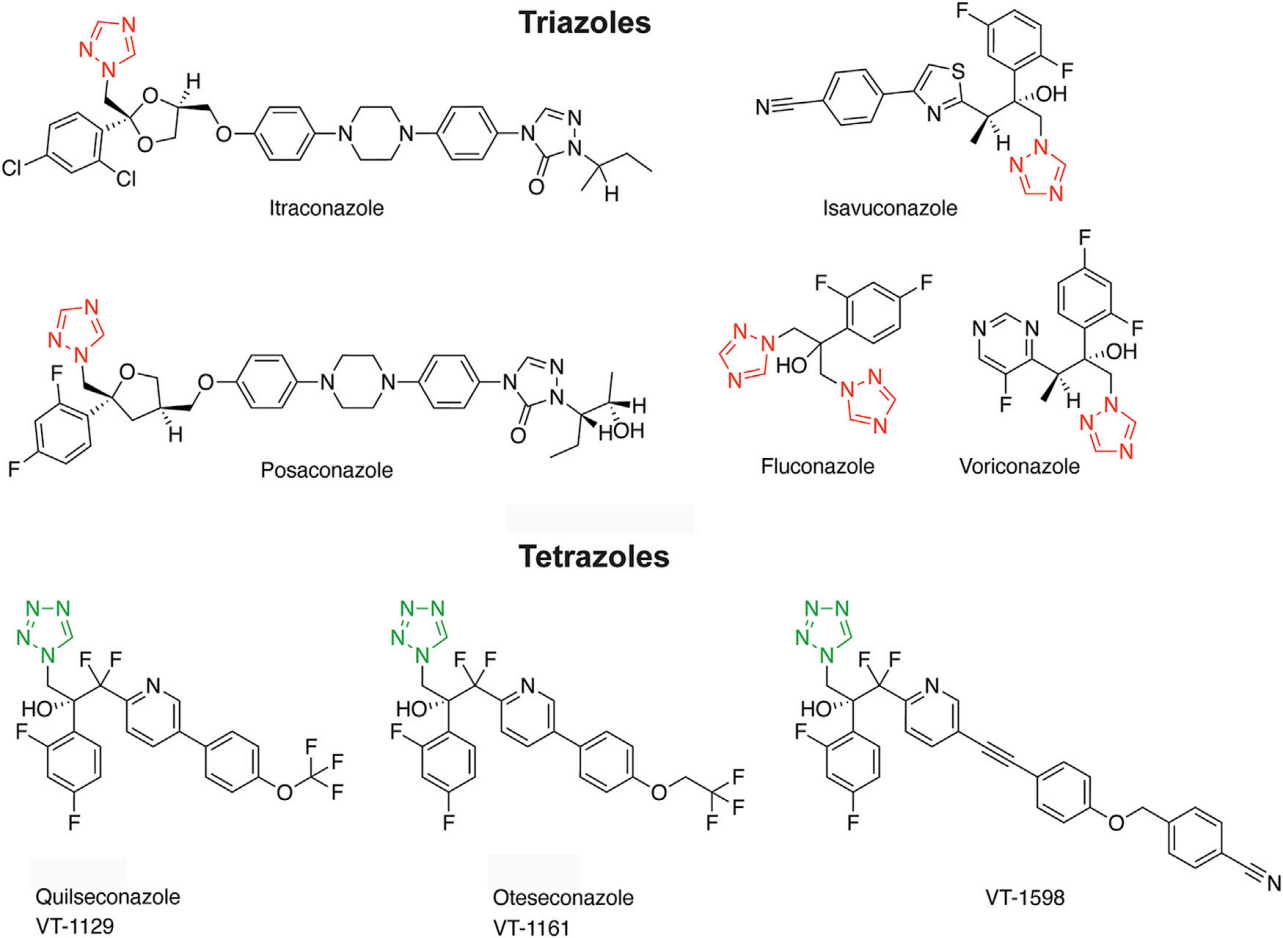
Structures of triazole and tetrazole antifungals with their characteristic aromatic heterocycle depicted in red and green, respectively.

Triazole antifungals are the most widely used first line therapy against fungal infections and they are applied for prophylactic treatment of immune compromised patients (Odds et al., 2003; Allen et al., 2015; Martinez-Matias and Rodriguez-Medina, 2018; Gintjee et al., 2020). They inhibit fungal lanosterol 14α-demethylase (cytochrome-P450 enzyme CYP51) and disrupt the cell membrane integrity by blocking ergosterol synthesis. Triazole antifungals approved for systemic treatment of fungal infections include voriconazole, fluconazole, itraconazole, posaconazole and isavuconazole (see Figure 1 for structures). They were optimized for a broader antifungal spectrum and fewer adverse health effects due to more specific interactions with fungal CYP51 than the initially developed imidazole antifungals, whose applications are now mostly restricted to topical treatment (Allen et al., 2015). Many of the adverse effects of imidazoles and, although less pronounced, of triazole antifungals are a result of non-intentional inhibition of human CYP enzymes (Van Tyle, 1984; Obach et al., 2006; Allen et al., 2015; Martinez-Matias and Rodriguez-Medina, 2018; Beck and Odermatt, 2021). Whilst drug metabolizing CYPs are included in the standard test battery to detect potential off-target effects in pre-clinical safety assessment, CYPs metabolizing endogenous bioactive molecules are usually not assessed (Urban et al., 2015). For example, CYPs involved in the biosynthesis of steroids regulate essential physiological processes and their dysregulation contributes to major diseases. Thus, CYPs and hydroxysteroid dehydrogenases (HSDs) required for steroidogenesis are relevant for the assessment of off-target effects.

**FIGURE 2:**
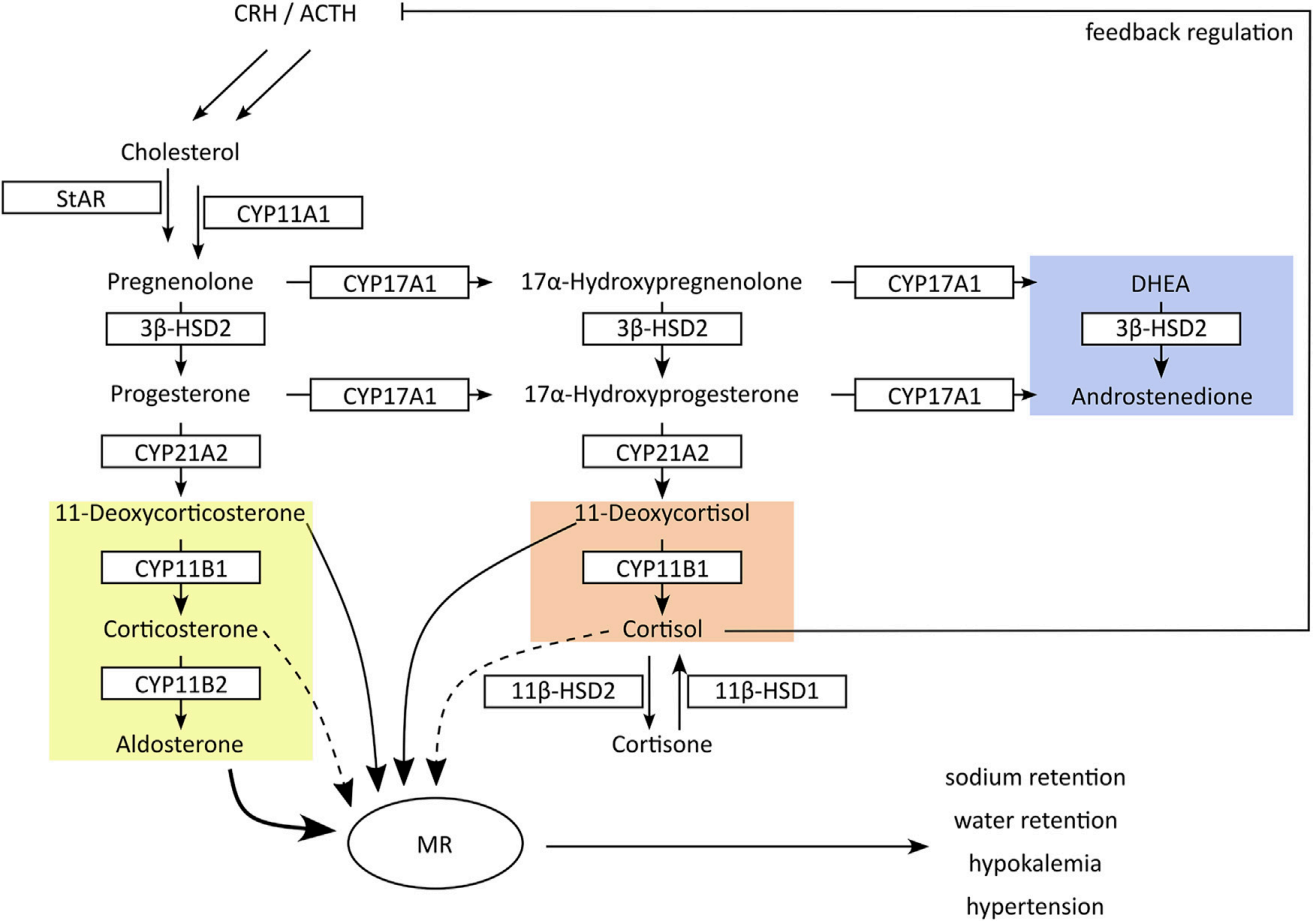
Schematic presentation of adrenal steroidogenesis. In the adrenals cytochrome-P450 (CYP) and hydroxysteroid dehydrogenase (HSD) enzymes are responsible for the synthesis of glucocorticoids (red), mineralocorticoids (yellow) and androgen precursors (blue). Inhibition of CYP17A1 17α-hydroxylase or CYP11B1 results in feedback stimulation of adrenal steroidogenesis to maintain circulating cortisol concentrations, accompanied by an excessive production of mineralocorticoids. In peripheral tissues, 11β-HSDs interconvert cortisol and its inactive metabolite cortisone. Inhibition of 11β-HSD2 in kidney results in cortisol-mediated activation of mineralocorticoid receptors (MR). Both mechanisms result in an excessive MR activation with hypertension and hypokalemia.

The adrenal glands produce three types of steroids: glucocorticoids, mineralocorticoids and androgen precursors (Figure 2) (Miller and Auchus, 2011). The rate-limiting step is the cholesterol transport to the intermembrane space of mitochondria via steroidogenic acute regulatory protein (StAR) and subsequent conversion to pregnenolone by the side-chain cleavage enzyme (CYPscc, CYP11A1). Their inhibition diminishes steroid output and induces compensatory mechanisms due to steroid insufficiency, thus posing a risk for adrenal hyperplasia (Miller and Auchus, 2011; Harvey, 2016). CYP17A1 converts pregnenolone and progesterone to their 17α-hydroxylated forms that can be further metabolized to the adrenal androgens dehydroepiandrosterone (DHEA) and androstenedione by the CYP17A1 17,20-lyase activity in the *zona reticularis*. Progesterone and 17α-hydroxyprogesterone are converted by CYP21A2 to mineralocorticoids and glucocorticoids in the *zona glomerulosa* and *zona fasciculata*, respectively.

Inhibition of 17α-hydroxylase reduces the production of adrenal androgens and glucocorticoids (*i.e.*, DHEA and cortisol) but not mineralocorticoids. Decreased cortisol levels then lead to the activation of the hypothalamic-pituitary-adrenal (HPA) axis with elevated release of corticotrophin releasing hormone (CRH) and adrenocorticotrophic hormone (ACTH), attempting to maintain physiological cortisol levels (Gjerstad et al., 2018). As a result, inappropriately high amounts of mineralocorticoids are produced, resulting in excessive mineralocorticoid receptor (MR) activation (Miller and Auchus, 2011; Beck et al., 2020a). CYP11B1 and CYP11B2 produce the terminal glucocorticoid cortisol and mineralocorticoid aldosterone, respectively. Due to the feedback response, inhibition of CYP11B1 results in an accumulation of the CYP21A2 products 11-deoxycorticosterone (11-DOC) and cortexolone (11-deoxycortisol), which are moderate MR agonists but at elevated levels can cause pseudohyperaldosteronism with hypertension and hypokalemia (Funder, 2007). Thus, the non-intentional inhibition of steroidogenic CYPs can lead to severe adverse health effects.

A major off-target effect due to CYP17A1 and CYP11B1 inhibition has been reported for the imidazole ketoconazole following systemic treatment of fungal infections (Pont et al., 1982; Santen et al., 1983; Pont et al., 1984; Aabo and De Coster, 1987; Wiederhold, 2018; Beck et al., 2020a). For these reasons, ketoconazole was replaced by triazole antifungals for systemic therapy. However, in recent years several studies revealed a risk of pseudohyperaldosteronism with hypertension and hypokalemia following treatment with itraconazole and posaconazole, with up to 25% of treated patients being affected (Nguyen et al., 2020; Beck and Odermatt, 2021). Other symptoms resulting from steroidogenic CYP inhibition after treatment with itraconazole or posaconazole include gynecomastia (Thompson et al., 2020), steroid insufficiency with hyperplasia (Skov et al., 2002; Molimard et al., 2008; Miller et al., 2018; Pimentel et al., 2018; Agarwal et al., 2020; Araque et al., 2020), and edema (Beck and Odermatt, 2021). Another off-target effect of the triazole antifungals itraconazole and posaconazole includes inhibition of 11β-HSD2 (27, 28), a member of the short-chain dehydrogenase/reductase (SDR) family like the steroidogenic enzyme 3β-HSD2. This abolishes glucocorticoid inactivation in peripheral tissues such as kidney and colon and results in cortisol-mediated MR activation with hypertension and hypokalemia (Beck et al., 2017; Thompson et al., 2017; Thompson et al., 2019; Beck and Odermatt, 2021).

To reduce the risk for adverse drug reactions, next-generation tetrazole antifungals were designed to increase the interactions with the substrate-binding pocket of fungal CYP51 and diminish the interaction with the ferric (Fe^3+^) iron of the porphyrin ring characteristic of all CYPs (Hoekstra et al., 2014; Gillardi and Di Nardo, 2017; Yates et al., 2017; Gintjee et al., 2020). The tetrazole antifungals analyzed in this study include quilseconazole (VT-1129), whose development was terminated, VT-1598 that recently entered clinical development (ClinicalTrials.gov, study NCT04208321), and oteseconazole (VIVJOA^®^, VT-1161) that was recently approved for treatment of vulvo-vaginal candidiasis (Hoy, 2022; Mycovia, 2022) (see Figure 1 for structures). In preclinical studies, oteseconazole and VT-1598 demonstrated more favorable MIC_50_ values (minimal inhibitory concentration reducing the fungal load by 50%) than the currently used systemic triazole antifungals, along with a broader antifungal spectrum (Shubitz et al., 2015; Lockhart et al., 2016; Warrilow et al., 2017; Wiederhold et al., 2018; Nishimoto et al., 2019a; Nishimoto et al., 2019b; Martens et al., 2022) (see Supplementary Table S1 for a comparison of selected MIC values). In addition, they showed only weak inhibitory activity against drug metabolizing CYPs, such as CYP3A4, CYP2C9, CYP2C19, thus exhibiting a highly reduced risk for drug-drug interactions compared with the triazole antifungals (Warrilow et al., 2014; Warrilow et al., 2016; Yates et al., 2017). However, despite favorable properties, their potential to inhibit steroidogenic CYPs or peripherally expressed steroid dehydrogenases, as recently demonstrated for the triazoles itraconazole and posaconazole (Beck and Odermatt, 2021), has not yet been assessed.

In the guidance for industry “Nonclinical Evaluation of Endocrine-Related Drug Toxicity,” the Food and Drug Administration (FDA) recommends to assess effects on CYP enzymes “involved in anabolism and catabolism of steroid hormones”; however, experimental procedures and endpoints are currently not defined (FDA, 2015). This study employed a recently established workflow based on an extended protocol of the Organisation for Economic Co-operation and Development (OECD) guideline 456 using H295R adrenocortical tumor cells (OECD, 2011; Strajhar et al., 2017; Akram et al., 2019), combined with activity assays using recombinant enzymes to characterize the mechanism of adrenal steroidogenesis perturbations (Jäger M. et al., 2023).

This work aimed to assess the potential of oteseconazole, VT-1598 and quilseconazole to disrupt adrenal steroidogenesis and compare their effects with those of the systemic triazole antifungals (Jäger M. et al., 2023). Culture supernatants of forskolin-stimulated H295R cells exposed to the tetrazole antifungals were subjected to an extended steroid profiling based on liquid-chromatography-high-resolution mass-spectrometry (LC-HRMS) in order to increase the information gain and compare steroidogenic footprints (Tonoli et al., 2015; Jäger et al., 2023b). Subsequent targeted quantification of nine adrenal steroids allowed estimating the risk for CYP11B1 and/or CYP17A1 inhibition as well as detecting other steroidogenesis disturbances. Direct enzyme inhibition was then tested using recombinant human adrenal steroidogenic enzymes. In addition, the tetrazoles were subjected to a cell-free 11β-HSD2 activity assay to assess a potential risk of causing pseudohyperaldosteronism.

## 2 Materials and methods

### 2.1 Chemicals and reagents

Prochloraz (CAS: 67747-09-5), posaconazole (CAS: 171228-49-2), itraconazole (CAS: 84625-61-6), ketoconazole (CAS: 65277-42-1), fluconazole (CAS: 86386-73-4), voriconazole (CAS: 137234-62-9), isavuconazole (CAS: 241479-67-4), bisphenol A (CAS: 80-05-7), 11-deoxycorticosterone (11-DOC, CAS: 64-85-7), [2,2,4,6,6,21,21,21-D8]-17α-hydroxyprogesterone (CAS: 850023-80-2), [2,2,3,4,4,6-D6]-dehydroepiandrosterone (CAS: 1261254-39-0), [2.2.4.4.6.6.21.21-D7]-aldosterone (CAS: 1261254-31-2), and [2,2,4,6,6-D5]-cortexolone were obtained from Sigma-Aldrich (Buchs, Switzerland). [1,2,6,7-3H]-cortisol (CAS: 50-23-7), and [1,2,6,7-3H(N)]-progesterone (CAS: 94391-12-5) were purchased from Perkin Elmer (Schwerzenbach, Switzerland), [1,2-3H]-cortisone (53-06-5) from American Radiolabeled Chemicals (St. Louis, MO). 17α-hydroxypregnenolone (CAS: 387-79-1), oteseconazole (VT-1161, CAS: 1340593-59-0), and 20S-hydroxycholesterol (20OH-cholesterol, CAS:516-72-3) were obtained from MedChemExpress (Monmouth Junction, United States). Glycyrrhetinic acid was purchased from Fluka (Charlotte, North Carolina, United States). Androstenedione (CAS: 63-05-8), dehydroepiandrosterone (DHEA, CAS: 53-43-0), corticosterone (CAS: 50-22-6), cortexolone (CAS:152-58-9), aldosterone (CAS: 52-39-1), cortisol (CAS: 50-23-7), progesterone (CAS: 57-83-0) and 17α-hydroxyprogesterone (CAS: 68-96-2) were purchased from Steraloids (Newport, RI, United States) and [2,2,6,6,17,21,21,21-D8]-progesterone (unlabeled CAS 57-83-0) from Cambridge isotope laboratories (Andover, MA, United States). Quilseconazole (VT-1129) and VT-1598 were a kind gift from Mycovia Inc. (Durham, NC, USA). Methanol (Biosolve, Dieuze, France) and dimethyl sulfoxide (DMSO, CAS: 67-68-5, Acros Organics, Geel, Belgium) was used to prepare stock solution. Solvents for ultra-high-performance-liquid-chromatography tandem mass-spectrometry (UHPLC-MS/MS) included ethyl acetate and acetonitrile and were ordered from Fisher Scientific (Reinach, Switzerland) and Scharlau (Barcelona, Spain), respectively.

### 2.2 Cell culture

Human Embryonic Kidney-293 cells (HEK293), V79-4, COS-1 and H295R cells were obtained from American Type Culture Collection (ATCC, Manassas, VA, United States). Stably transfected V79-CYP11B1 cells were a kind gift from Dr. Rita Bernhard (Denner et al., 1995a; Denner et al., 1995b). V79-4, V-79-CYP11B1 and COS-1 cells were cultivated in Dulbecco`s modified Eagles`s medium (DMEM, Sigma-Aldrich) supplemented with 4 mg/mL glucose, 4 mM L-glutamine, 100 U/mL penicillin/streptomycin (Sigma-Aldrich), and 10% (v:v) fetal bovine serum (FBS, Biowest, Nuaillé, France). H295R cells were cultured in DMEM/Ham`s nutrient mixture F12 (1:1, v:v) (Live Technologies, Zug, Switzerland) mixed with 1% ITS + Premix (BD Bioscience, Bedford, MA, United States), 2.5% (v:v) Nu-serum (Lot: 0154001, BD Bioscience), 15 mM 4-(2-hydroxyethyl)-1-piperazineethanesulfonic acid (HEPES) buffer (pH 7.4), and 100 U/mL penicillin/streptomycin. HEK293 cells were cultured in DMEM containing 10% FBS (v:v), 100 U/mL penicillin/streptomycin, 2 mM L-glutamine, 10 mM HEPES, pH 7.4, 10% MEM non-essential amino acids (v:v), and 4.5 g/L glucose.

### 2.3 H295R steroidogenesis assay and cell viability test in H295R cells

The H295R steroidogenesis assay was performed as recommended by the OECD test guideline 456 (OECD, 2011) with modifications described earlier (Jäger M. et al., 2023). Cells of passage 5 to 10 were seeded (200,000 cells/mL) and incubated for 24 h. Then, compounds of interest (0.03 µM, 0.1 µM, 0.3 µM, 1 μM, and 3 µM) and forskolin (10 µM) were added to the cells, followed by incubation for 48 h. Complete medium served as time zero control. Cell culture supernatant was stored at −20°C until preparing samples for steroid quantification by UHPLC-MS/MS. Cell viability in the presence of substances of interest was determined as described earlier using the 2,3-bis/2-methoxy-4-nitro-5-sulfophenyl)-2H-tetrazolium-5-carboxanilide (XTT) assay (Jäger M. et al., 2023). No effect on cell viability by any of the compounds and concentrations applied could be observed.

### 2.4 Determination of CYP11A1 activity

CYP11A1 activity was measured as described earlier (Jäger M. et al., 2023). Briefly, V79-4 cells (3,000,000) were seeded in 10 cm dishes and transfected with 8,000 ng DNA containing equal amounts of expression plasmid for CYP11A1 and adrenodoxin using polyethylemine (PEI) in a PEI/DNA ratio of 3:1 (w/w). Mitochondria were prepared as described previously (Jäger et al., 2023c). For the activity assay, 500 μg/mL of mitochondrial fraction were preincubated with 1 mM NADPH, and either 0.1% DMSO vehicle, ketoconazole as positive control (Mast et al., 2013) or the compound of interest. After 10 min the reaction was started by adding 20S-hydroxycholesterol at a final concentration of 2 µM in a final volume of 50 µL. The reaction was incubated for 4 h at 37°C and stopped with 50 µL methanol. Samples were evaporated to dryness, reconstituted in 6 µL of methanol, diluted with water 1:25 (v:v) and analyzed using a pregnenolone enzyme-linked immunosorbent assay (ELISA) kit according to the manufacturer (Alpco, 11-PREHU-E01, Salem, United States).

### 2.5 Determination of CYP11B1 activity

CYP11B1 activity was determined as described earlier (Jäger et al., 2023c). Briefly, 500 μg/mL of mitochondrial fraction protein of V79 cells stably expressing CYP11B1 and 10 μM of the test compound were incubated in reaction buffer for 10 min. The reaction was started by adding 1 mM NADPH and 11-DOC at a final concentration of 500 nM. The final reaction volume was 50 μL. The reaction was incubated for 90 min at 37°C in a thermoshaker at 300 rpm. Liquid nitrogen was used to stop the reaction. Samples were stored at −80°C until steroid quantification using UHPLC-MS/MS.

### 2.6 CYP17A1 and CYP21A2 activity assays

CYP17A1 and CYP21A2 activities were measured as described recently (Jäger M. et al., 2023), using microsomal fractions of transfected COS-1 cells. For CYP17A1, cells were cotransfected with plasmids for CYP17A1 and P450-oxoreductase (POR) using PEI. 17α-hydroxylase activity was determined in the presence of 500 nM progesterone containing 10 nM radiolabeled progesterone, 1 mM NADPH, in the absence or presence of the respective azole antifungal. After reaction termination, 17α-hydroxyprogesterone formation was determined by separating substrate and product by thin-layer chromatography (TLC), excising steroid bands, and scintillation counting. CYP17A1 17,20-lyase activity was measured using microsomal preparations of COS-1 cells cotransfected with plasmids for CYP17A1, POR and cytochrome b5, followed by incubation in the presence of 500 nM 17α-hydroxypregnenolone, 1 mM NADPH, in the absence or presence of the respective azole antifungal. DHEA formation was detected using an ELISA kit (Demeditec GmbH, Kiel, Germany).

For CYP21A2 activity, microsomal preparations of COS-1 cells transfected with PEI and plasmids for CYP21A2 and POR were incubated with 500 nM progesterone containing 10 nM radiolabeled progesterone, 1 mM NADPH, in the absence or presence of the respective azole antifungal. Steroids were separated by TLC, followed by excision of the steroid bands and analysis of 11-DOC formation by scintillation counting.

### 2.7 11β-HSD and 3β-HSD2 activity assays

11β-HSD1 and 11β-HSD2 activities were measured as described earlier (Kratschmar et al., 2011; Beck et al., 2017). HEK293 cells stably expressing 11β-HSD1 and hexose-6-phosphate dehydrogenase or 11β-HSD2 were lysed and the lysates stored at −80°C until usage. For 11β-HSD1 activity, lysates were incubated with 200 nM radiolabeled cortisone, 500 µM NADPH and compounds of interest at 37°C for 10 min and shaking at 300 rpm in a total volume of 22 µL. For 11β-HSD2 activity, 200 nM radiolabeled cortisol and 500 µM NAD^+^ were used. After the incubation, an excess amount of substrate and product (1:1, 2 mM each, in methanol) was added to the reaction. Steroids were separated via TLC using chloroform and methanol (9:1, v:v) and product formation was determined relative to the substrate by scintillation counting.

3β-HSD2 was measured in lysates of HEK293 cells as described previously (Jäger M. et al., 2023). Briefly, HEK293 cells transiently expressing 3β-HSD2 were harvested in ice-cold phosphate buffered saline. Cells were centrifuged at 4°C at 16,000 × g for 4 min, shock-frozen in liquid nitrogen and stored at −80°C. For enzyme activity measurements, cell pellets were lysed with 10 sonication pulses of an ultrasonic probe and diluted to obtain about 10 ng/mL progesterone from 500 nM pregnenolone in 1 h. Cell lysate was incubated with 10 µM of the substance of interest in TS2 buffer (100 mM NaCl, 1 mM ethylene glycol-bis(2-aminoethylether)-N,N,N′,N′-tetraacetic acid (EGTA), 1 mM ethylenediaminetetraacetic acid (EDTA), 1 mM MgCl_2_, 250 mM sucrose, 20 mM tris(hydroxymethyl)aminomethane (Tris) HCl, pH 7.4) for 8 min before starting the reaction by adding 1 mM NAD+ and 500 nM pregnenolone. After 1 h of incubation at 37°C, the reaction was stopped by heat inactivation for 1 min at 95°C. Progesterone formation was determined using an ELISA kit (DE1516, Demeditec).

### 2.8 Targeted steroid quantification using UHPLC-MS/MS

Concentrations of steroid hormones present in the cell culture supernatant of H295R cells were determined using UHPLC-MS/MS as described previously (Jäger M. et al., 2023). Briefly, H295R samples (450 µL) were spiked with aldosterone-D7, cortexolone-D5, corticosterone-D8, 17-hydroxyprogesterone-D8 and progesterone-D8 to final concentrations of 0.25 ng/mL each, and DHEA-D6 to a final concentration of 1 ng/mL. The total sample volume was adapted to 1 mL using Milli-Q water. Samples were extracted with Oasis HLB 3 cc cartridges (30 mg, 30 µm particle size) preconditioned with ethyl acetate and Milli-Q water. Samples were loaded and washed with 1 mL methanol (10%, v:v) and Milli-Q water before steroid elution with 2 × 750 µL ethyl acetate. Samples were divided in two parts and evaporated to dryness. One-half of the sample was reconstituted in 50 µL methanol, the other was stored at −80°C until untargeted analysis.

The workflow to determine concentrations of 11-DOC and corticosterone present in reaction volumes of CYP11B1 activity measurements is described elsewhere (Jäger et al., 2023c). Briefly, for solid-phase extraction (SPE), each sample was mixed with protein precipitation solution (100 μL, 0.8 M zinc sulfate in water/methanol; 50:50, v:v), the internal standard corticosterone-D8 to a final concentration of 0.25 ng/mL, and Milli-Q water to a final volume of 1 mL. Cartridges were preconditioned with methanol and Milli-Q water prior to sample loading. Samples were washed with 10% and 40% methanol (v:v in Milli-Q water) and eluted with twice 750 µL 100% methanol. After evaporation to dryness, samples were reconstituted in 500 µL methanol and diluted 1:4 using methanol. Eluted samples were applied to a reverse phase column (Waters Acquity UPLC BEH C18, 1.7 µm, 2.1 mm × 150 mm) and separated using an Agilent 1290 UHPLC system (Agilent Technologies, Basel, Switzerland) and two mobile phases consisting of acetonitrile, water and formic acid (95:5:0.1; v:v:v and 5:95:0.1; v:v:v). Steroids were detected with an Agilent 6495 triple quadrupole mass spectrometer with a jet stream ionization source. For data analysis Mass Hunter software version B.09.00 (Agilent Technologies) was used. In case the signal to noise was below five, calculations were performed using lower limit of detection (LLOD) divided by two. LLOD and lower limit of quantification (LLOQ) concentrations are listed in Supplementary Table S2.

### 2.9 Untargeted steroid profiling and data analysis

Untargeted steroid profiling was performed as described (Jäger et al., 2023b). Samples were extracted and evaporated as outlined above, and reconstituted in 50 µL methanol:water (1:1; v:v). A quality control pool (QC) was prepared by mixing 9 µL of each sample. QCs were injected at the beginning of the analytical sequence in order to equilibrate the instrument, and at regular intervals to monitor analytical performance during the analytical batch (Pezzatti et al., 2020). UHPLC-MS/MS steroid analysis was conducted as previously described (Randazzo et al., 2017; Codesido et al., 2019). Briefly, chromatography was performed on a C18 core-shell column (Kinetex C18 100 $\overset{{}^{\circ}}{A}$ , 2.1 × 150 mm, 1.7 µm, Phenomenex, Torrance, United States) fitted with the corresponding pre-column, and using a gradient profile from 2% to 100% of B in 14 min. Mobile phases A and B were H_2_O with 0.1% formic acid (FA) and acetonitrile with 0.1% FA respectively. A Waters H-Class Acquity system (Waters) coupled to a maXis 3G Q-TOF high resolution mass spectrometer (Bruker Daltonik GmbH, Bremen, Germany) through an electrospray interface operated in positive ionization mode were used for the analyses. HyStar v3.2 SR2 software (Bruker Daltonik) with Waters Acquity UHPLC v1.5 plug-in was used for instrument control and data acquisition. Run alignment, peak-picking and annotation were performed on Progenesis QI v2.3 (Nonlinear Dynamics, Waters, Newcastle upon Tyne, United Kingdom). Steroid annotation and identification was conducted by comparing their accurate masses, retention times, and fragmentation patterns when available, to those of standard compounds analyzed under the same conditions, to the level of confidence indicated in Supplementary Table S3.

### 2.10 Multivariate analysis

Multivariate models were computed using SIMCA 17 (Sartorius Stedim Data Analytics AB, Umeå, Sweden). Unit variance scaling was used as pre-processing. Principal Component Analysis (PCA) was first applied to assess the samples distribution and the detection of the major trends in the dataset without prior information on experimental conditions. Orthogonal Partial Least Squares regression (OPLS (Trygg and Wold, 2002)) was then used to separate variations that are useful for predicting the dependent response from other sources of orthogonal variations uncorrelated to it (Supplementary Table S4). This supervised method is an extension of standard PLS that was applied to extract salient patterns characterizing the different classes (discriminant analysis). Model validity was assessed using 7-fold cross-validation. Interpretation was further aided by a visualization tool allowing straightforward comparisons of metabolic patterns, namely, the Shared and Unique Structure (SUS) plot (Wiklund et al., 2008). It can be used to relate the contributions of metabolites to different OPLS models by taking advantage of the predictive components. This makes it possible to distinguish variables with similar patterns in different models, or to highlight situation-specific signatures.

## 3 Results

### 3.1 Grouping of azole antifungals and steroid footprinting

To assess whether oteseconazole, VT-1598 and quilseconazole interfere with adrenal steroidogenesis, we applied a previously established untargeted steroidomics approach (Jäger et al., 2023b) and compared steroid levels in supernatants of H295R cells stimulated with forskolin and treated with various concentrations of systemic triazole and tetrazole antifungals. This allowed contrasting steroid footprints and obtaining information on concentration-dependent effects. Samples for the analysis of triazole antifungal-induced effects on adrenal steroidogenesis were prepared previously (Jäger M. et al., 2023). Because voriconazole and fluconazole did not substantially affect adrenal steroidogenesis at the lower concentrations, only the two highest concentrations (3 μM and 1 µM) were included for comparison in this study. For the other azole antifungals 3 μM, 1 μM, and 0.3 µM were assessed. No cytotoxic effects were observed for the compounds and concentrations applied.

**FIGURE 3:**
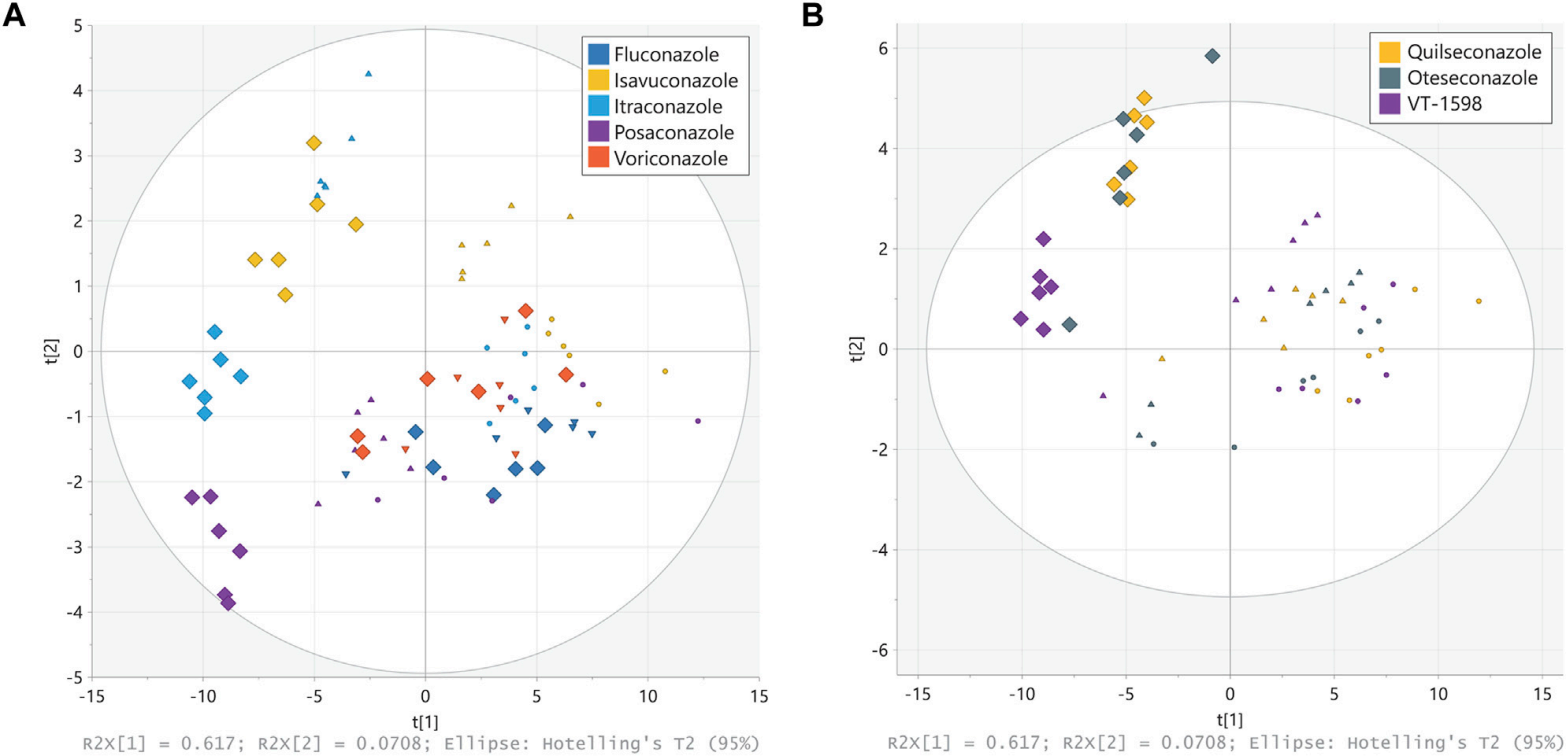
Principal component analysis (PCA) of steroid patterns obtained from H295R cells. H295R cells were treated with different concentrations of triazole **(A)** and tetrazole **(B)** antifungals (rectangles displays 3 µM treatment, triangles 1 µM and circles 0.3 µM). Cell culture supernatants were analyzed using an untargeted LC-HRMS approach with steroid annotation and identification. Experiments were performed three times independently, each in duplicate. Results were normalized to the DMSO control. Individual experimental results appear as one point on the PCA scores plot.

The untargeted LC-HRMS analysis with subsequent metabolite annotation and identification resulted in the assignment of 63 stable analytical features corresponding to putative or confirmed steroids. Principal component analysis (PCA) was carried out to offer a first insight into the most salient variability patterns of the measured steroid profiles. The first principal plane summarizing 62% (PC1) + 7% (PC2) of the total variability clearly distinguished effects on steroid profiles triggered by treatment with different triazole antifungals (Figure 3A). Moreover, it revealed a similar but slightly different pattern for voriconazole and fluconazole, in line with observations from a previous study showing negligible effects of the two compounds on the levels of nine steroids measured by targeted UHPLC-MS/MS (Jäger M. et al., 2023). A clear separation was obtained for the groups corresponding to the highest posaconazole, itraconazole and isavuconazole concentrations. The steroid patterns obtained from treatments with 1 μM and 0.3 µM of posaconazole, itraconazole and isavuconazole formed distinct clusters, even though their separation was less pronounced than at 3 µM. In contrast, the patterns derived from the 1 μM and 0.3 µM tetrazole concentrations could not be clearly distinguished on this PCA score plot (Figure 3B). The tetrazole antifungal induced effects were only prominent for the highest treatment concentrations, with a clear separation of VT-1598 from oteseconazole and quilseconazole (Figure 3B). Samples from exposure to oteseconazole and quilseconazole at 3 µM were grouped in the same area of the score plot as is the case after treatment with 1 µM of itraconazole, suggesting similar levels of most measured steroids. To further study steroid patterns and potential differences between more specific alterations, supervised multivariate models using OPLS regression were generated for each azole using their respective concentration levels as response vector. All models were found significant using cross-validation with Q^2^ values >0.7. Full details of OPLS models are available in Supplementary Table S4. Then, SUS plots were used for graphical interpretation using pairwise comparisons enabling to detect discriminant and common patterns in their steroidogenesis inhibitory profiles (Supplementary Figure S1). For example, the levels of hydroxylated progestin metabolites were affected in a more pronounced manner by oteseconazole (3 µM) than itraconazole (1 µM) (Supplementary Figure S1B). This helped differentiate the effects caused by oteseconazole and itraconazole, which present specific characteristics although being globally similar. In addition, the SUS plot suggested that itraconazole suppressed the levels of several hydroxylated androgen and estrogen metabolites, while oteseconazole treatment of H295R cells appeared to increase their concentrations (Supplementary Figures S1A, B). The PCA plot from the 3 µM treatments with VT-1598 and isavuconazole indicated very similar steroid footprints. Nevertheless, the SUS plot suggested that selected progestin and estrogen metabolites could show a difference since they were suppressed in a slightly more pronounced manner by VT-1598 than isavuconazole (Supplementary Figures S1C, D).

### 3.2 Assessing a potential risk of pseudohyperaldosteronism for tetrazole antifungals using the H295R cell model and targeted steroid quantification

A first goal of this work was to evaluate the risk of pseudohyperaldosteronism for tetrazole antifungals. This became especially interesting because in the PCA scores plot the tetrazoles oteseconazole and quilseconazole (3 µM) showed an overall similar pattern to that of 1 µM itraconazole that is known to cause pseudohyperaldosteronism by inhibiting adrenal CYP11B1 and peripheral 11β-HSD2 (Beck et al., 2020b; Beck and Odermatt, 2021).

**FIGURE 4:**
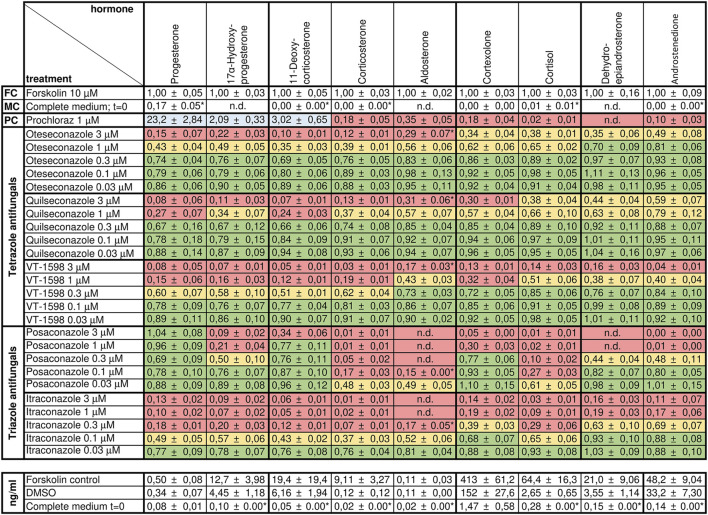
Effects of selected tetrazole antifungals on the steroid profile of forskolin-stimulated H295R cells. Forskolin-stimulated H295R cells were incubated with the positive control prochloraz or different concentrations of oteseconazole, quilseconazole and VT-1598 for 48 h. Steroid concentrations in cell culture supernatants were determined by UHPLC-MS/MS. Experiments were performed three times independently, each in duplicate. Data were normalized to the forskolin control (FC) and fold changes are shown as mean ± SD. *n.d.* indicates steroids for which no peak could be observed. LLOD/2 was used for calculations when the signal to noise was below five (indicated with an asterisk, see Supplementary Table S1 for method sensitivity). Color-coding indicates percent reduction in steroid formation relative to forskolin with ≥67% remaining steroid formation depicted in green, 33%–66% in yellow, and ≤32% in red. Values ≥ 200% are highlighted in blue.

We analyzed supernatants from H295R cells incubated with forskolin and different concentrations of tetrazole antifungals by targeted quantification of nine adrenal steroids using targeted UHPLC-MS/MS. DMSO served as vehicle control, forskolin as induction control, and prochloraz, as recommended by the OECD guideline 456 (Hecker et al., 2011), as adrenal steroidogenesis inhibitor (Figure 4). The same controls as in the previous experiments on triazole antifungals were used (Jäger M. et al., 2023). All three tetrazole antifungals significantly suppressed steroid biosynthesis in a concentration-dependent manner. The steroid patterns did not reveal an impaired production of a specific steroid. Oteseconazole showed moderate inhibitory effects at 1 µM with approximately 60% lower 11-DOC and corticosterone formation and approximately 40% inhibition of aldosterone, cortexolone, and cortisol formation relative to the forskolin control (Figure 4; Supplementary Figure S2). Quilseconazole and VT-1598 showed a very similar pattern, with VT-1598 tending to be more potent. VT-1598 inhibited the formation of 11-DOC and corticosterone by 50% and 40% at 300 nM, respectively, and by about 80%–90% at 1 µM. Effects on aldosterone, cortexolone and cortisol were less pronounced with 60%, 70%, and 50% inhibition at 1 μM, respectively.

To estimate the ability of the tetrazole antifungals to inhibit aldosterone and cortisol production, relative to that by posaconazole and itraconazole (Beck et al., 2020b; Beck and Odermatt, 2021), the results from the present study were compared with those from earlier results on triazoles (Jäger M. et al., 2023). This direct comparison indicated a much weaker impact of the tetrazole antifungals compared to posaconazole and itraconazole (Supplementary Figure S2). Aldosterone formation was completely inhibited by 100 nM posaconazole and 300 nM itraconazole, while tetrazole antifungals inhibited aldosterone output by 20%–30% at 300 nM (Supplementary Figure S2A). Complete inhibition after tetrazole treatment was only seen at 3 µM. Effects on cortisol output were most pronounced with posaconazole, leading to 40% inhibition after H295R cell treatment with 30 nM (Supplementary Figure S2B). For tetrazole antifungals, a similarly potent inhibition was only observed at concentrations ≥1 µM.

A previous study showed that product to substrate ratios provide more reliable information on the inhibition of a specific enzyme than individual steroid levels (Jäger et al., 2023b). Importantly, while cortisol/cortexolone and corticosterone/11-DOC, indicative of CYP11B1 activity, showed concentration-dependent decreases for posaconazole and itraconazole, the three selected tetrazoles did not lower these ratios, suggesting no substantial inhibition of the enzyme (Supplementary Figure S3). Also the 17α-hydroxyprogesterone/progesterone ratio suggested that none of tetrazoles inhibits CYP17A1 17α-hydroxylase activity (Supplementary Figure S3).

### 3.3 Enzyme activity assays to assess the potential of the selected tetrazole antifungals to cause pseudohyperaldosteronism

**FIGURE 5:**
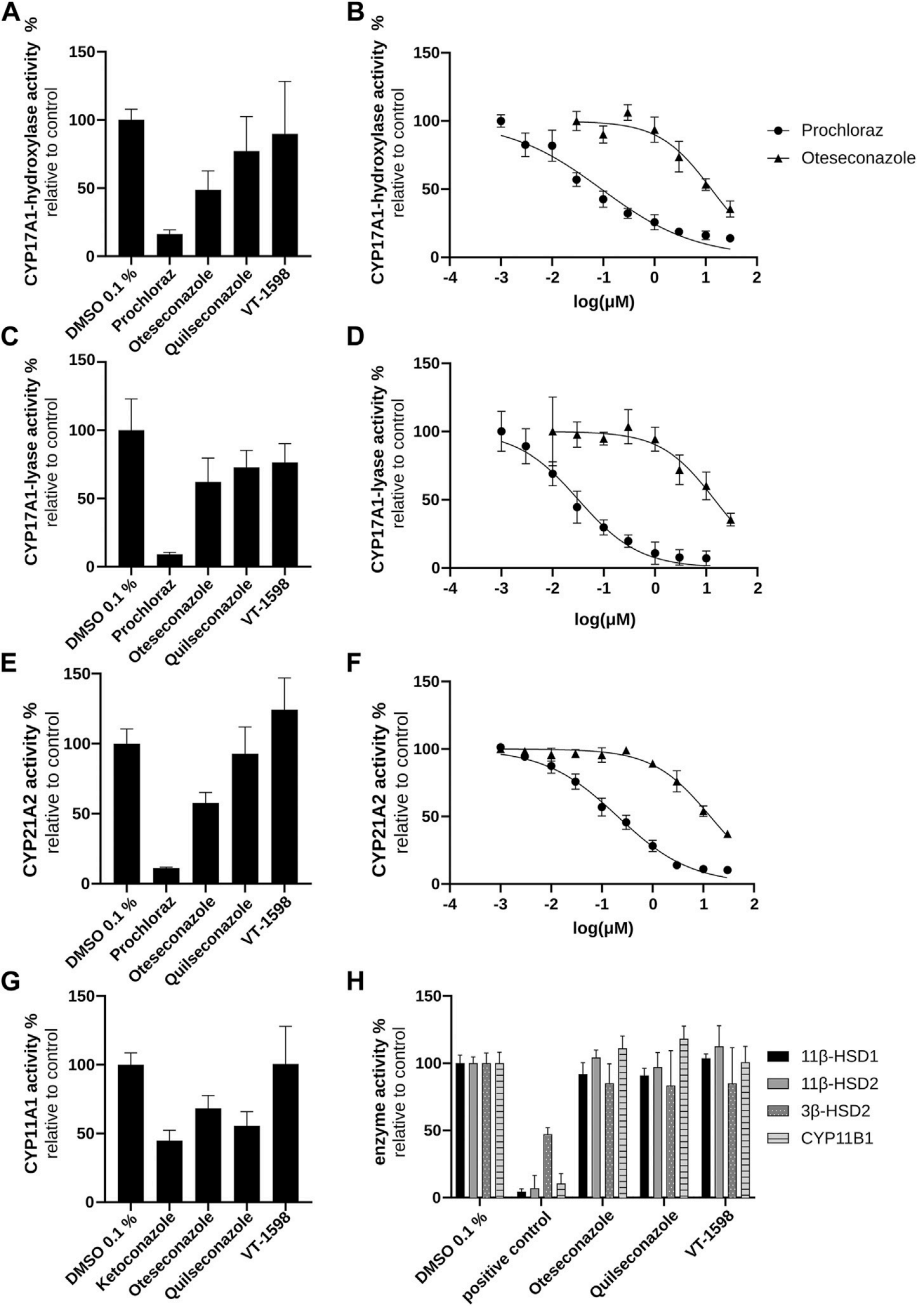
Inhibitory effects of selected azole antifungals on CYP11B1, CYP17A1, CYP21A2, 11βHSD1, 11βHSD2, and 3βHSD2. Initial screenings were performed at 10 µM of the tested substances. CYP17A1 and CYP21A2 activities were determined using microsomal fractions of transfected COS-1 cells, measuring the formation of 17α-hydroxyprogesterone from progesterone (CYP17A1 17α-hydroxylase reaction) **(A, B)**, DHEA from 17α-hydroxypregnenolone (CYP17A1 17,20-lyase reaction) **(C, D)** and 11-DOC from progesterone (CYP21A2) **(E, F)**. For the CYP11A1 activity assay, the formation of pregnenolone from 20α-hydroxycholesterol was measured using mitochondrial fractions of transfected V79-4 cells **(G)**. CYP11B1 activity was assessed using mitochondrial preparations of transfected V79-4 cells and measuring the conversion of 11-DOC to corticosterone, with ketoconazole serving as positive control. Activities of 11β-HSDs and 3β-HSD2 were measured using lysates of transfected HEK293 cells. Cortisol and cortisone were quantified as substrate and product for 11βHSD2 and the reverse reaction for 11βHSD1 activity. Progesterone was quantified as product of the 3β-HSD2 catalyzed reaction with pregnenolone as substrate. Glycyrrhetinic-acid and bisphenol A were used as positive controls to inhibit 11β-HSDs and 3β-HSD2, respectively **(H)**. Experiments were performed at least three times independently. Results were normalized to the DMSO control and represent mean ± SD.

To verify the conclusion drawn above based on the H295R experiments, cell-free CYP11B1 activity measurements were performed. No direct inhibition of CYP11B1 activity by the tetrazoles could be observed (Figure 5H), supporting the conclusions from the product/substrate ratios (Supplementary Figure S3). The cell-free 17α-hydroxylase activity assay revealed a weak inhibition for oteseconazole (IC_50_ value of 13.2 ± 3.9 µM; Figures 5A,B), whereas quilseconazole and VT-1598 did not inhibit (Figure 5A).

Next, experiments using lysates of HEK293 cells stably expressing 11β-HSD2 or 11β-HSD1 (to address the reverse reaction) were performed. Neither 11β-HSD2 nor 11β-HSD1 activity was affected by the selected tetrazole antifungals (Figure 5H). Thus, the enzymes involved in pseudohyperaldosteronism, namely, CYP11B1, CYP17A1 17α-hydroxylase and 11β-HSD2, were not inhibited by the three tetrazoles.

### 3.4 Tetrazole-mediated inhibition of steroidogenesis by mechanisms not associated with pseudohyperaldosteronism

In H295R cells, the tested tetrazole antifungals reduced overall steroidogenic output, with a slightly more pronounced effect observed for VT-1598 compared to oteseconazole and quilseconazole (Figure 4). As shown above, this effect is independent of CYP11B1 and CYP17A1 17α-hydroxylase activity. An overall reduced steroidogenesis in H295R cells was previously reported for itraconazole and isavuconazole, the latter slightly inhibiting CYP11A1 activity (Jäger M. et al., 2023). Based on the PCA results highlighting the major trends in the data, the overall similar footprints of VT-1598 and isavuconazole could hint on a VT-1598-dependent CYP11A1 inhibition. Its inhibition is expected to decrease the concentration of its direct product pregnenolone and all downstream steroids. To test whether the tetrazoles inhibit CYP11A1, a cell-free assay using mitochondrial preparations of V79-4 cells transiently expressing adrenodoxin and CYP11A1 was applied. The positive control ketoconazole, previously reported as potent CYP11A1 inhibitor (Loose et al., 1983; Mast et al., 2013), showed about 55% inhibition at 10 µM (Figure 5G). This may either indicate that ketoconazole is not a potent CYP11A1 inhibitor, or that the applied assay had limited sensitivity, allowing a rather qualitative assessment of CYP11A1 inhibition. Nevertheless, quilseconazole and oteseconazole inhibited CYP11A1 by about 45% and 35%, respectively, at 10 μM, whereas VT-1598 was inactive at this concentration. Because VT-1598 showed more potent suppression of overall steroid production in H295R cells than oteseconazole and quilseconazole, a mechanism distinct from direct CYP11A1 inhibition must be responsible for this observation (Figure 4). The reduced overall steroid output upon exposure to the selected tetrazoles (Figure 4) could be a result of 3β-HSD2 inhibition, the enzyme converting pregnenolone, 17α-hydroxypregnenolone and DHEA to progesterone, 17α-hydroxyprogesterone and androstenedione, respectively. The cell-free activity assay using lysates of transfected HEK293 cells revealed that the selected tetrazoles do not inhibit 3β-HSD2 (Figure 5H). CYP21A1 catalyzes the formation of 11-DOC and cortexolone from progesterone and 17α-hydroxyprogesterone, respectively (Figure 2), and its inhibition represents a risk for adrenal hyperplasia (Miller and Auchus, 2011). To see whether inhibition of CYP21A2 plays a role in the reduced overall steroidogenesis in H295R cells, a cell-free enzyme activity assay using microsomal preparations from COS-1 cells was performed. Whilst VT-1598 and quilseconazole did not exhibit an inhibitory activity, oteseconazole moderately inhibited CYP21A2, with an IC_50_ value of 13.8 ± 1.9 µM (Figures 5E,F).

Of the three tetrazole antifungals, only VT-1598 has been studied for inhibition of CYP17A1 17, 20-lyase, with an IC_50_ value about 1000 times higher than that for posaconazole (Yates et al., 2017). In line with these observations, the present study found very weak inhibitory effect for VT-1598. Among the three tetrazoles, oteseconazole was the most potent compound with an IC_50_ value of 14.8 ± 3.0 µM (Figures 5C,D).

### 3.5 Revisiting steroid measurements in patients from a clinical phase 2 study on oteseconazole

To begin to assess possible disturbances in steroid levels in patients, previously unpublished results on steroid measurements from a clinical phase 2 study in patients suffering from onychomycosis and treated with 600 mg oteseconazole for 12 or 24 weeks (Elewski et al., 2021) were revisited. In this earlier study, the plasma concentrations of DHEA, androstenedione, testosterone, progesterone and its 17α-hydroxylated metabolite were determined by HPLC-MS/MS (see Supplementary information for method details) but the values had not been published. Corticosteroids were not measured. As shown in Supplementary Table S5, none of these steroids significantly differed between placebo control and treated groups, *i.e.*, after 12 or 24 weeks of administration of 600 mg oteseconazole, or between treatment and post-treatment follow-up at 60 or 96 weeks. Moreover, the gonadotropins luteinizing hormone and follicle-stimulating hormone, measured by ELISA, were unaffected by oteseconazole treatment (Mycovia Pharmaceuticals Inc., personal communication).

## 4 Discussion

The results of this study confirmed the hypothesis that tetrazole antifungals exhibit a low risk to disturb adrenal steroidogenesis by inhibiting steroidogenic CYPs. Using our recently established extended H295R assay using targeted UHPLC-MS/MS-based quantification, we detected a concentration-dependent decrease of all nine adrenal steroids measured. Although aldosterone, cortisol and 17α-hydroxyprogesterone production was decreased (Figure 4; Supplementary Figure S2), which might suggest inhibition of the pseudohyperaldosteronism targets CYP11B1 and CYP17A1, the respective product to substrate ratios were not altered (3 μM) (Supplementary Figure S3). This indicates that CYP11B1 and CYP17A1 activities are not substantially affected by direct inhibition or downregulation of gene expression following the exposure of the H295R cells to the tetrazoles. This emphasizes the value of including product to substrate ratios for obtaining initial mechanistic information on a compound’s effect on steroidogenesis using the H295R assay. In support of this interpretation, the cell-free enzyme activity assays revealed that oteseconazole, quilseconazole and VT-1598 did not inhibit CYP11B1. With respect to CYP17A1, oteseconazole, but not the other two tetrazoles, showed a weak inhibitory effect against CYP17A1 17α-hydroxylase activity in the cell-free assay (13.2 ± 3.9 μM); however, this was not enough to alter the 17α-hydroxyprogesterone/progesterone ratio marker in H295R cells.

The weak CYP17A1 inhibition detected in the *in vitro* experiments unlikely results in hypertension in patients. In fact, the overall rate of reports of hypertension adverse events in subjects treated with oteseconazole in a 36-week study was not higher (6/212 = 3%) than in those on placebo (2/47 = 4%) (Elewski et al., 2021), and neither 17α-hydroxyprogesterone nor the 17α-hydroxylase activity marker 17α-hydroxyprogesterone/progesterone ratio (Medeiros et al., 2013) decreased upon treatment of patients with 600 mg oteseconazole (Supplementary Table S5). Nevertheless, to fully exclude an effect through CYP17A1 hydroxylase and subsequent adaptation by feedback regulation, the concentrations of ACTH as well as renin and aldosterone should be determined in patients (Gjerstad et al., 2018). Potent 17α-hydroxylase inhibition by the prostate cancer drug abiraterone leads to impaired cortisol production with subsequent feedback activation, increased ACTH levels, accumulation of the mineralocorticoids 11-DOC and corticosterone, water retention, hypertension, and hypokalemia (Attard et al., 2012). However, it needs to be noted that the inhibitory potency of abiraterone against CYP17A1 is 2–3 orders of magnitude higher than that of oteseconazole.

In addition, the three tetrazole antifungals did not inhibit the glucocorticoid inactivating enzyme 11β-HSD2. Thus, contrary to the triazoles itraconazole and posaconazole (Beck et al., 2020b), oteseconazole, quilseconazole and VT-1598, unlikely cause pseudohyperaldosteronism by inhibiting CYP11B1, CYP17A1 17α-hydroxylase and 11β-HSD2 activities (Miller and Auchus, 2011; Beck et al., 2020a; Beck et al., 2020b). Itraconazole is clearly the most potent 11β-HSD2 inhibitor amongst the systemic triazole fungicides (Beck et al., 2020b) and, to our knowledge, is the only azole antifungal reported to induce peripheral edema (Diaz et al., 1991; Lestner et al., 2009; Antonarakis et al., 2013; Lee et al., 2019; Teaford et al., 2020). Interestingly, the licorice constituent glycyrrethinic acid, a potent 11β-HSD2 inhibitor, was also reported to induce peripheral edema (Stormer et al., 1993; Deutch et al., 2019). Thus, 11β-HSD2 inhibition might explain the observed itraconazole-induced peripheral edema.

Interestingly, the tetrazoles as well as itraconazole and isavuconazole blocked steroidogenesis in H295R cells. The steroid patterns obtained for the three tetrazoles looked very similar when nine adrenal steroids were analyzed. However, untargeted steroidomics, covering steroids that may be either produced *de novo* by the H295R cells or added by the serum and further metabolized by various enzymes expressed in these cells, confirmed this overall observation but also allowed detecting more subtle differences between the compounds tested. This is thought to be a valuable first step to identify any changes in the steroidome due to exposure. Footprinting and chemical grouping based on omics data is increasingly accepted, with the OECD working on a reporting framework to simplify omics-generated toxicological data submission (Harrill et al., 2021). Using a common protocol should eventually lead to a database of steroid patterns for different chemicals. In this project, untargeted steroidomics analyses of supernatants from H295R cells exposed to triazole and tetrazole antifungals allowed comparing their effects. Voriconazole and fluconazole grouped together, while posaconazole, itraconazole and isavuconazole clearly showed a different overall pattern, in line with a recent study assessing only nine adrenal steroids (Jäger M. et al., 2023). Comparison of the steroidogenic footprints showed that oteseconazole and quilseconazole were comparable. Their patterns from the highest concentration (3 µM) resembled that of the intermediate itraconazole concentration (1 µM). The steroid profile derived from VT-1598 shared some similarity with that of isavuconazole. Despite globally similar steroid footprints, SUS plots allowed differences between oteseconazole and itraconazole, and between VT-1598 and isavuconazole to be highlighted. This qualitative assessment indicated discrepancies between oteseconazole (3 µM) and itraconazole (1 µM) as revealed by the production of hydroxylated progestins. In addition, several hydroxylated androgens and estrogens were decreased following exposure to itraconazole but enhanced by oteseconazole. Furthermore, VT-1598 lowered progestin and estrogen metabolites levels compared with isavuconazole. Many of the metabolites detected by the steroidomics method are derived from the Nu-serum added for culturing H295R cells. Since this study focused on interferences of azole antifungals with steroids produced by adrenal cells, effects on gonadal steroidogenesis and liver-derived metabolism need to be investigated in a follow-up study.

In an attempt to explain the differences in the steroid patterns of H295R cells for the compounds tested, we assessed effects on the remaining steroidogenic enzymes. The differences in the patterns from posaconazole and itraconazole may be explained, at least in part, by the potent inhibition of the CYP17A1 17, 20-lyase activity by posaconazole, resulting in less efficient downstream metabolism of progesterone and 17α-hydroxyprogesterone to DHEA and androstenedione, respectively. Oteseconazole weakly inhibited CYP17A1 17, 20-lyase activity, with a 50 times lower potency compared to posaconazole. Inhibition of the 17, 20-lyase activity was proposed as a contributing factor for posaconazole-induced gynecomastia (Thompson et al., 2020). Quilseconazole and VT-1598 exhibited even weaker 17, 20-lyase inhibitory potency than oteseconazole, suggesting a very low risk by the tetrazoles to cause adverse effects by lowering adrenal androgen levels. The levels of DHEA and androstenedione, as well as those of luteinizing hormone and follicle-stimulating hormone, were unaffected in male patients treated with 600 mg oteseconazole for 12 or 24 weeks in the recent phase II clinical study (Elewski et al., 2021) (Supplementary Table S5, personal communication with Mycovia Pharmaceuticals Inc.). Since potential effects on estrogen concentrations in female patients were not yet analyzed, follow-on studies should assess possible inhibitory effects on aromatase and estrogen biosynthesis to evaluate a potential disturbance of estrogen balance.

The weak inhibition of CYP21A2 by oteseconazole and lack of effect on 3β-HSD2 suggest a negligible risk of adrenal insufficiency and hyperplasia via this mechanism (Miller and Auchus, 2011). To our knowledge, no cases of adrenal insufficiency and hyperplasia due to oteseconazole treatment have been reported so far, and plasma DHEA concentrations were unaffected in patients treated with 600 mg daily for 12 or 24 weeks in a recent phase II study (Supplementary Table S5). This is different for the potent CYP11B1 inhibitors posaconazole and itraconazole where cases with adrenal insufficiency and hyperplasia have been described, mainly due to cross-reactions with other drugs depending on functional CYP3A4 or in combination with unrelated diseases (Skov et al., 2002; Molimard et al., 2008; Miller et al., 2018; Pimentel et al., 2018; Araque et al., 2020). Since oteseconazole, quilseconazole and VT-1598 exhibit weak inhibitory effects on drug metabolizing CYP enzymes (Warrilow et al., 2016; Warrilow et al., 2017; Yates et al., 2017), their potential for cross-reactions with other drugs can be considered low.

Furthermore, the general steroidogenesis block is unlikely explained by CYP11A1 inhibition. A weak CYP11A1 inhibition was observed for oteseconazole and quilseconazole and, as reported earlier, for isavuconazole (Jäger M. et al., 2023), VT-1598, despite being about three times more efficient in blocking steroidogenesis, did not inhibit CYP11A1. As the steroidogenesis block seen in H295R cells must involve an early step in steroidogenesis, follow-up-studies should address whether expression and activity of StAR, the rate-limiting step of cholesterol transfer into the mitochondria, or cholesterol supply are affected. In patients, a reduced overall adrenal steroid output is usually compensated via activation of the HPA axis in response to low circulating cortisol levels and characteristically elevated ACTH release. Prolonged exposure to high ACTH levels (long term drug treatment or Cushing’s disease) results in adrenal cellular hypertrophy and hyperplasia, with increased expression of StAR and steroidogenic enzymes in an attempt to maintain circulating cortisol levels (Miller and Auchus, 2011). Drug-induced CYP11A1 inhibition was documented for aminoglutethimide, with increased ACTH release and plasma renin levels (Fishman et al., 1967). So far, ACTH levels have not been determined in patients treated with isavuconazole or the tetrazoles. Thus, it is unclear whether a feedback-mediated mechanism might compensate for potentially reduced steroid levels *in vivo*. Given the unchanged androgen levels in male patients treated with oteseconazole (Supplementary Table S5), it seems possible that concentrations causing the observed *in vitro* effect are not reached *in vivo* or the moderate inhibition is compensated by feedback regulation. Future studies should measure mineralocorticoid, glucocorticoid and ACTH levels to assess potential compensatory effects on adrenal steroidogenesis.

The patient’s relevance of the steroidogenesis block observed in H295R cells for itraconazole (at 0.3 μM = 0.21 μg/mL), isavuconazole (at 1 μM = 0.44 μg/mL), oteseconazole (at 3 μM = 1.58 μg/mL) and VT-1598 (at 1 μM = 0.58 μg/mL), given it is granted permission for clinical use, depends on the concentration reached within the adrenals. Clinical experience with the azole antifungals itraconazole and posaconazole showed high inter-individual variability of plasma drug levels (Andes et al., 2009; Dolton et al., 2014; Dekkers et al., 2016), and the risk for pseudohyperaldosteronism correlated with plasma drug concentrations (Beck and Odermatt, 2021). The recommended drug plasma levels for oteseconazole (C_min_ 2.5 μg/mL, C_max_ 2.8 μg/mL for the highest treatment concentration (Mycovia, 2022)) are in the range of recommendations for isavuconazole (C_max_ 2.6 μg/mL), posaconazole (C_min_ 1 μg/mL, C_max_ 3 μg/mL (Beck and Odermatt, 2021) or 3.75 μg/mL (Cornely et al., 2016; Cornely et al., 2017)) and slightly exceed those for itraconazole (C_max_ 2.0 μg/mL for the highest treatment concentration (Janssen-Pharmaceuticals-Inc, 2001)). Considering drug binding to serum proteins, itraconazole, posaconazole, isavuconazole and oteseconazole are similar, with 98%-99% serum protein binding. Thus, the expected free drug concentrations are about 50-fold lower (Janssen-Pharmaceuticals-Inc, 2001; Bellmann and Smuszkiewicz, 2017; Chen et al., 2020; Mycovia, 2022). As intra-tissue concentrations can exceed those in plasma, it is difficult to estimate the concentrations reached in the adrenals. A main difference with respect to pharmacokinetic properties is the drug half-life, which is about 30 h for itraconazole and posaconazole, 80–120 h for isavuconazole, and 138 days for oteseconazole (Janssen-Pharmaceuticals-Inc, 2001; Bellmann and Smuszkiewicz, 2017; Chen et al., 2020; Mycovia, 2022). In contrast to the triazole antifungals, oteseconazole was found to be poorly metabolized (Mycovia, 2022) and, to our knowledge, no data on major metabolites of VT-1598 and quilseconazole have been reported so far. While a longer half-life might increase the risk for adverse effects due to longer accumulation periods, it may also increase the stability of drug plasma levels due to less frequent administration of the drug (Smith et al., 2018).

In conclusion, oteseconazole, VT-1598 and quilseconazole exhibit a negligible risk of causing pseudohypoaldosteronism as they neither inhibited the adrenal enzymes CYP11B1 and CYP17A1 needed for cortisol production nor the peripherally expressed 11β-HSD2 that prevents excessive glucocorticoid-dependent MR activation. At the highest concentrations tested (*i.e.* 3 μM and 1 µM) the three tetrazole antifungals caused an overall inhibition of steroidogenesis in the H295R adrenal cell model, an effect most pronounced for VT-1598. However, these concentrations are still an order of magnitude higher than those reported for posaconazole-dependent CYP11B1 inhibition and itraconazole-dependent 11β-HSD2 inhibition, respectively, that are responsible for causing pseudohyperaldosteronism in patients reaching high plasma levels of these drugs (mostly exceeding the envisaged C_max_ values). Despite the expected low risk of the tetrazole antifungals and isavuconazole to interfere with steroid synthesis, the mechanism underlying the observed inhibition of overall steroid synthesis should be elucidated in follow-up experiments, in the context of avoiding adrenal insufficiency and hyperplasia.

## Data Availability

The original contributions presented in the study are included in the article/Supplementary Material or are publicly available. This data can be found here: Figure 3 and Supplementary Figure S1 (DOI: 10.5281/zenodo.13133369; URL: https://doi.org/10.5281/zenodo.13133369). Figure 4 (DOI: 10.5281/zenodo.13133380; URL: https://doi.org/10.5281/zenodo.13133380). Figure 5 and Supplementary Figure S2. (DOI: 10.5281/zenodo.13133961; URL: https://doi.org/10.5281/zenodo.13133961).
