# Supplementary material for: Assessment of the potential risk of oteseconazole and two other tetrazole antifungals to inhibit adrenal steroidogenesis and peripheral metabolism of corticosteroids
Source: Front Pharmacol. 2024 Aug 8;15:1394846. doi: 10.3389/fphar.2024.1394846 (PMC11338861; doi:10.3389/fphar.2024.1394846)
Supplement: Supplementary file 1 [file DataSheet1.zip › Supplementary materials.DOCX]

## **Supplementary material**

**Methods**

**Sample preparation and HPLC-MS/MS measurement to determine androgen concentrations in plasma samples from male patients**

Plasma samples were collected from male patients who participated in the previously published phase II study (59). Steroid concentrations and details on the method were provided by Mycovia Pharmaceuticals.

Stable isotopic labelled internal standards for each analyte were added to study samples, calibration standards, and quality control samples. The analytes and their internal standards were extracted by liquid-liquid extraction using methyl tertiary-butyl ether (MTBE) from all samples (200 µL, or sample). The MTBE extracts were evaporated to dryness then reconstituted in methanol:water (80:20). The steroids in the reconstituted sample were separated by using reversed-phase liquid chromatography with detection of the eluate by tandem mass spectrometric detection (HPLC-MS/MS) using Agilent Jet Stream electrospray ionization in the positive ion mode. HPLC-MS/MS data were acquired using the proprietary software application MassHunter Workstation Data Acquisition for Triple Quad B.07.01 (Build 7.1.7112.0), Agilent Technologies, Inc.). Data were processed (integrated) using the software application MassHunter Quantitative Analysis for QQQ (version B.07.01/Build 7.1.524.0. Agilent Technologies, Inc.). Calibration plots of area ratio versus steroid concentrations were constructed and the steroid-specific model was applied to the data using MassHunter Quantitative Analysis for QQQ.

**Table S1: Direct comparisons of minimal inhibitory concentrations (MIC_50_) for some triazole and investigated tetrazole antifungals published elsewhere.** MIC_50_ are given in µg/mL. References are listed in brackets.

| MIC_50_ values µg/mL | *Coccidioides immitis* | *Coccidioides posadasii* | *Candida albicans* | Candida glabrata | Cryptococcus neoformans | *Trichophyton rubrum* |
| --- | --- | --- | --- | --- | --- | --- |
| Posaconazole |  |  | 0.5 (38) | < 0.03 – 2 (39) |  |  |
| Itraconazole |  |  | 0.25 (38) | 0.125 - 2 (39) |  | 0.06 (33) |
| Fluconazole | 8 (34), 16 (35) | 8 (34), 16 (35) | 0.25 (36) | 2 – 64 (39), 2 (36) | 2 (37) |  |
| Oteseconazole | 2 (34) | 1 (34) | 0.125 (38)  0.004 (36) | < 0.015 – 1 (39)  0.03 (36) |  | <0.03 (33) |
| Quilseconazole |  |  |  |  | < 0.015 (37) |  |
| VT-1598 | 0.5 (35) | 1 (35) | 0.06 (38) | < 0.015 – 1 (39) |  |  |

**Table S2: Sensitivity of the UHPLC-MS/MS for quantified steroids.** Concentrations are given in ng/mL.

|  | Progesterone | 17α-hydroxy-progesterone | 11-DOC | Cortico-sterone | Aldosterone | Cortexolone | Cortisol | DHEA | Androstene-dione |
| --- | --- | --- | --- | --- | --- | --- | --- | --- | --- |
| LLOD | 0.02 | 0.20 | 0.09 | 0.03 | 0.03 | 0.09 | 0.56 | 0.30 | 0.28 |
| LLOQ | 0.06 | 0.34 | 0.28 | 0.19 | 0.06 | 0.28 | 0.99 | 0.81 | 0.60 |

**Table S3: Steroid annotation.** Steroid annotation and identification was conducted by comparing their accurate masses, retention times, and fragmentation patterns when available, to those of standard compounds analyzed under the same conditions, to the level of confidence indicated.

See uploaded Table S3 csv file.

**Table S4: OPLS regression models with corresponding values of R^2^X, R^2^Y, and Q^2^.**R^2^X = proportion of variance explained in the predictor variables, R^2^Y = goodness of fit, Q^2^Y = goodness of prediction.

| **OPLS Model** | **Model size** | **R^2^X** | **R^2^Y** | **Q^2^Y** |
| --- | --- | --- | --- | --- |
| Isavuconazole | 1 pred + 2 ortho | 0.774 | 0.979 | 0.902 |
| Itraconazole | 1 pred + 2 ortho | 0.798 | 0.983 | 0.944 |
| Oteseconazole | 1 pred + 1 ortho | 0.770 | 0.816 | 0.706 |

**Table S5: Steroid concentrations measured in human plasma samples of male patients treated with placebo or oteseconazole for 12 or 24 weeks.** Concentrations were determined but not published in the course of a phase II study published previously (59). Steroid levels were determined using HPLC-MS/MS (see method description in Appendix above for further details). *reflects removal of an outlier value.

|  |  |  | Progesterone  ng/dL | | 17OH-Progesterone ng/dL | | Androstenedione  ng/mL | | Testosterone  ng/dL | | DHEA  ng/mL | |
| --- | --- | --- | --- | --- | --- | --- | --- | --- | --- | --- | --- | --- |
| Otese-conazole | Week | Patient Count | Average | SD | Average | SD | Average | SD | Average | SD | Average | SD |
| placebo | 2 | 18 | 5.2 | 3.3 | 48 | 23 | 0.59 | 0.29 | 371 | 134 | 1.86 | 1.45 |
|  | 12 | 21 | 6 | 4.5 | 52 | 37 | 0.59 | 0.32 | 362 | 174 | 1.37 | 0.74 |
|  | 24 | 23 | 4.4 | 3.3 | 43 | 22 | 0.56 | 0.34 | 359 | 134 | 1.18 | 0.68 |
|  | 60 | 21 | 5.7 | 4.5 | 46 | 22 | 0.52 | 0.19 | 452 | 336 | 0.91 | 0.58 |
|  | 96 | 19 | 5.2 | 4 | 39 | 20 | 0.48 | 0.22 | 348 | 156 | 1.14 | 0.62 |
| 600 mg  12 weeks | 2 | 13 | 4.3 | 3.9 | 54 | 41 | 0.71 | 0.39 | 305 | 123 | 1.38 | 1.26 |
|  | 12 | *20 | 5.2 | 5.6 | 53 | 36 | 1.12 | 0.78 | *368 | *182 | 1.69 | 1.37 |
|  | 24 | 20 | 4 | 4.9 | 48 | 25 | 1.04 | 0.65 | 379 | 186 | 1.46 | 1.02 |
|  | 60 | 19 | 4.8 | 4.3 | 59 | 44 | 0.99 | 0.73 | 374 | 203 | 1.16 | 1.16 |
|  | 96 | 19 | 5.5 | 6.3 | 62 | 49 | 1.04 | 0.85 | 336 | 163 | 1.53 | 1.53 |
| 600 mg  24 weeks | 2 | 17 | 4.1 | 2.7 | 46 | 23 | 0.85 | 0.37 | 351 | 123 | 1.82 | 1.03 |
|  | 12 | 21 | 3.9 | 2.8 | 44 | 20 | 1.04 | 0.41 | 427 | 209 | 1.5 | 1.05 |
|  | 24 | 21 | 3.3 | 2.1 | 37 | 19 | 1.03 | 0.48 | 365 | 156 | 1.64 | 0.97 |
|  | 60 | 22 | 3.6 | 3.1 | 44 | 35 | 1.03 | 0.57 | 357 | 141 | 1.54 | 0.96 |
|  | 96 | 19 | 4.4 | 3.4 | 46 | 36 | 0.94 | 0.56 | 333 | 151 | 1.41 | 0.76 |

**Figure S1: SUS plots comparing effects of the treatment of H295R cells with different azole antifungals on the steroid metabolite profile based on OPLS regression predicting concentration exposure.** (A) itraconazole (1 µM) *vs.* oteseconazole (3 µM) (B) Zoom of the lower left part of figure A (marked rectangle). (C) Isavuconazole *vs.* VT-1598 (D) Zoom of the lower left corner of C (marked rectangle). Cells were treated for 24 hours and metabolites were analyzed using untargeted LC-MS. (to enlarge see additionally uploaded PDF file of Fig. 1S).


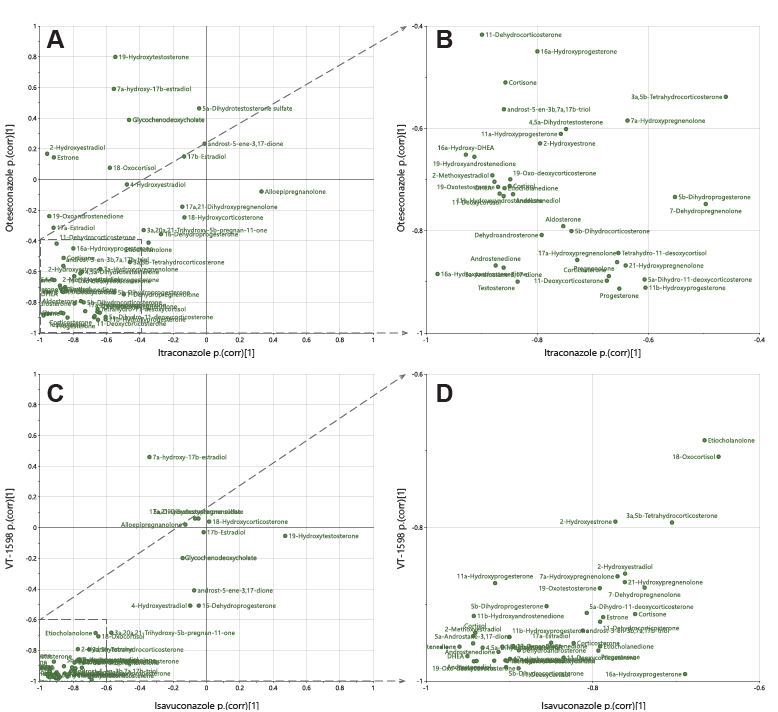


**Figure. S2. Inhibition of aldosterone and cortisol biosynthesis by tetrazole antifungals compared to posaconazole and itraconazole.** H295R cells were incubated for 48 hours with different concentrations of selected azole fungicides and the steroid concentrations in the supernatants were analyzed using UHPLC-MS/MS. Concentration-dependent inhibition of aldosterone (A) and cortisol (B) production is shown. LLOD/2 was used for calculations when aldosterone could not be reliably quantified. Posaconazole and itraconazole values were from a previous study (49). Results represent mean ± SD from three independent experiments.





**Figure S3: Product to substrate ratios indicative of CYP11B1 (A and B) and CYP17A1 17α-hydroxylase (C) activity after treatment with triazole and tetrazole antifungals.** Forskolin-stimulated H295R cells were incubated for 48 hours with various concentrations of azole antifungals. Steroid concentrations in cell culture supernatants were quantified by UHPLC-MS/MS and concentrations normalized to those of the forskolin control. Experiments were performed three times independently and ratios are presented as mean ± SD.
